# Manatee calf call contour and acoustic structure varies by species and body size

**DOI:** 10.1038/s41598-022-23321-7

**Published:** 2022-11-15

**Authors:** Beth Brady, Eric Angel Ramos, Laura May-Collado, Nelmarie Landrau-Giovannetti, Natalija Lace, Maria Renee Arreola, Gabriel Melo Santos, Vera Maria Ferreira da Silva, Renata S. Sousa-Lima

**Affiliations:** 1 Mote Marine Laboratory, Sarasota, FL USA; 2 The Rockefeller University, New York, NY USA; 3 Fundación Internacional para la Naturaleza y la Sustentabilidad, Chetumal, Quintana Roo Mexico; 4 University of Vermont, Burlington, VT USA; 5 Smithsonian Tropical Research Institute, Panama City, Panama; 6 Caribbean Manatee Conservation Center, Inter American University of Puerto Rico, Bayamón, PR USA; 7 Cetalingua Project, Tampa, FL USA; 8 Dolphin Discovery, Puerto Aventuras, Quintana Roo Mexico; 9 Biology and Conservation of Amazonian Aquatic Mammals, Federal Rural University of the Amazon, Belém, Pará Brazil; 10 Ecology Department, Rio de Janeiro State University, Rio de Janeiro, Rio de Janeiro Brazil; 11 Laboratory of Aquatic Mammals, National Institute of Amazonian Research, Manaus, Amazonas Brazil; 12 Laboratory of Bioacoustics, Department of Physiology and Behavior, Biosciences Center, Federal University of Rio Grande do Norte, Natal, Rio Grande do Norte Brazil

**Keywords:** Ecology, Evolution

## Abstract

Vocal activity and signal characteristics of mammals are driven by several factors that result in both stability and plasticity over multiple time scales. All three extant species of manatee communicate with several calls that are especially important for maintaining contact between cows and calves. Determining if calf calls differ across manatee species will provide insights into the evolution of species-specific acoustic communication traits. We investigated the interspecific differences in the vocalizations of calves of Amazonian manatees (*Trichechus inunguis*) and the two subspecies of the West Indian manatee (*T. manatus*). Vocalizations of individual calves were recorded in rehabilitation centers in Brazil, Puerto Rico, the United States, and Mexico. The acoustic structure of calls produced by manatee calves varied between species and with body size. Amazonian manatee calves produced shorter calls with multiple notes at higher frequency while West Indian calves produced modulated calls that were lower in frequency and longer in duration. Smaller West Indian calves produced frequency modulated, hill-shaped calls that flattened with an increase in body length. Our results provide evidence for divergence in the ontogeny of vocalizations across *T. manatus* and *T. inunguis* and suggest variation in body size contributed to the evolution of differences in the characteristics of their calls.

## Introduction

Vocal behavior is a key modality in animal communication in which different evolutionary pressures and ecological factors influence changes in the acoustic structure of calls^1,2^. Vocal variation within related species can be the result of genetic differences, geographic separation, and effects of habitat type^3,4^. On a species-specific scale, the structure of vocalizations can vary with behavior (e.g., African elephants, *Loxodonta africana*)^5^, between individuals for identification (bottlenose dolphins, *Tursiops truncatus*)^6^, during ontogeny (e.g., harbor seal pups, *Phoca vitulina*^7^; meerkats, *Suricata suricata*)^8^, be genetic and/or learned^9^ and influenced by body size^10,11^.

The size and length of the vocal tract and vocal apparatus influences the acoustic features of a signal and may be a reliable indicator of body size^10,11^. The production of vocalizations in vertebrates can be described according to the two-stage process of the source filter theory^12^. The source-filter theory predicts the signal is generated by the vibration of the laryngeal folds and subsequently filtered in the supralaryngeal vocal tract^12^. The length of the vocal folds determines the minimum fundamental frequency of a vocalization and has an allometric relationship with body size^13^. Therefore, larger animals would have longer vocal folds and as a result have a lower fundamental frequency. Determining the factors that influence how communication systems evolve demands more study with taxa whose acoustic communication and the basic function of their acoustic calls are still poorly understood^14^. Such is the case with Sirenians, where we have limited knowledge about acoustic communication both within and amongst species.

Sirenians include three extant species of manatees: the West Indian manatee (*Trichechus manatus*)—divided into the Antillean manatee (*T. m. manatus*) and Florida manatee (*T. m. latirostris*) subspecies^15^, the Amazonian manatee (*T. inunguis*), the African manatee (*T. senegalensis*) and the dugong (*Dugong dugon*). All sirenians produce vocalizations^16–19^, but here we focus on comparisons within the West Indian and Amazonian manatee species due to their shared evolutionary paths and ecological contexts. Manatees in the Western Hemisphere (West Indian and Amazonian) inhabit tropical and subtropical coasts in the Americas. Amazonian manatees inhabit freshwaters throughout the Amazon River Basin of northern South America^20^. The Antillean manatee range includes Central America, northern South America, and the Caribbean^21^. Florida manatees are found throughout the southern United States with periodic sightings in Cuba^22^ and Mexico^23^. Both Florida and Antillean manatees inhabit salt, fresh and brackish environments^24^. The Antillean and Amazonian manatee species overlap in Brazil where both species live in sympatry but show low hybridization rates suggesting these species occupy different ecological niches, have evolved mechanisms of reproductive isolation, or hybrid offspring viability is low^25^.

In all manatee species, the only known stable association is the 1½—2 years of cow/calf dependency^26,27^. Calves are thought to be nutritionally independent by one year of age, but stay with their mother beyond this age, presumably to learn the location of warm water refuges (for Florida manatees) and food sources from cows^28,29^. Calves tend to be more vocal and vocalize about 2–6 times more frequently than their mothers^17,19,26,30,31^, although no significant difference in vocalization rate was found between calves and adults in Antillean manatees^18^. Calves produce vocalizations under a variety of conditions which include, traveling, during approach from conspecifics, when separated and prior to nursing^19^. Calf vocalizations have been described as ranging from tonal vocalizations with harmonics to atonal vocalizations^17–19,30,32^. Amazonian and Antillean calf calls contain signature information which may be useful in recognition between cow/calf pairs^17,18^.

Currently, there are no published studies investigating if there are differences in frequency and temporal parameters among calf species. A few studies compared vocalizations between West Indian^33^ and African manatee species^34^ and noted significant overlap in frequency and temporal parameters. Although these differences were observed, it is unclear if calls from the West Indian and African manatees were obtained from adults and/or calves. Amazonian manatees are the smallest in size of all manatee species and have a fundamental frequency range from 1 to 8 kHz^17^. This range is higher than the reported frequency parameters of other manatee species. Furthermore, Antillean and Amazonian manatees are smaller in body size than Florida manatees^20^ and it is unclear if there would be differences in frequency and temporal vocal parameters between populations based on body size. The source of manatee vocalizations is the larynx and sound vibrations are produced by forcing air through their thick vocal folds^35^. The smaller body size and subsequently the smaller length of the vocal folds of the Amazonian manatee could place physiological constraints on signal production throughout development and therefore affect signal transmission^36^.

In addition to a lack of information on differences in vocalizations across manatee populations, there are few studies investigating if and how calls change during development^31^. Manatees tend to grow rapidly from birth. In the first year of life, captive Antillean manatee calves gained an average of 106 kg and grew an average of 83 cm^37^. Research on Amazonian and Antillean manatees suggests that the fundamental frequency becomes more defined as the calf grows to a more adult-type call^17,18^. In Florida manatees, hill-shaped calls (high squeaks) are correlated with calf presence, but the study could not attribute calls to younger or older calves^32^. Evidence of the ontogeny of acoustic structure in calls is limited to the study of one individual Amazonian manatee. They found an Amazonian manatee calf produced calls with decreasing fundamental frequencies during the first year of life^31^. After the first year, recordings from this and five other individuals recorded during the transition from calf to juvenile showed vocalizations increased in frequency^31^.

Identifying the factors that shaped the acoustic structure of manatee calls across species and over the course of their ontogeny will facilitate a better understanding of their communication and vocal repertoire. Here, we investigated if the frequency and temporal variables of the vocalizations of manatee calves varies across species as a function of body size. Manatee vocalizations were compared using spectrographic analysis to identify if shifts in vocal parameters occur depending on species. Since body size can be correlated with fundamental frequency^10,11^, we would predict the smaller body size of the Amazonian manatee would lead to higher frequency parameters than the Florida and Antillean manatee.

## Methods

### Data collection

Data for this study were collected from captive manatee calves (n = 18) from two different species at eight different facilities in the USA (*n* = 1), Mexico (*n* = 1), and Brazil (*n* = 6) (Table 1). Except for the calf from Mexico and one calf in Brazil (Ere), all calves were born in the wild and were either orphaned or brought in with their mothers for rehabilitation. In all locations, acoustic recordings of manatee calf vocalizations were collected from healthy animals. The straight-line body length—a straight-line measure from the tip of the snout to the medial notch of the fluke—of each manatee calf was obtained using a measuring tape during routine health assessments by veterinary staff at each of the respective facilities^38^.

**Table 1:** Information on the manatee calves recorded in this study. The body length of each calf was measured during routine veterinary procedures according to standard protocols at each facility^38^ (*ZT* ZooTampa, *CMCC* Caribbean Manatee Conservation Center, *DD* Dolphin Discovery, *CMA* Centro Mamíferos Aquáticos/IBAMA, *INPA* Laboratório de Mamíferos Aquáticos of the Instituto Nacional de Pesquisas da Amazônia, *MPEG* Parque Zoobotânico of the Museu Paraense Emílio Goeldi, *CPPMA* Centro de Preservação e Pesquisa de Mamíferos Aquáticos da Manaus Energia, *BioMA* Biologia e Conservação de Mamíferos Aquáticos da Amazônia).

Country	State	Site name	Species	Subspecies	Individual	Body length (cm)	*n* calls analyzed
USA	Florida	ZT	West Indian	Florida	Agua	132.0	11
USA	Florida	ZT	West Indian	Florida	Emerald	128.0	10
USA	Florida	ZT	West Indian	Florida	Camlee	204.0	10
USA	Florida	ZT	West Indian	Florida	Furbie	205.0	11
USA	Florida	ZT	West Indian	Florida	Neuson	192.0	10
USA	Florida	ZT	West Indian	Florida	Muddy Barron	175.0	11
USA	Puerto Rico	CMCC	West Indian	Antillean	Aramaná	106.0	10
USA	Puerto Rico	CMCC	West Indian	Antillean	Guamá	128.0	12
Mexico	Quintana Roo	DD	West Indian	Antillean	BonBon	115.0	4
Brazil	Pernambuco	CMA	West Indian	Antillean	Guaju	199.0	10
Brazil	Pernambuco	CMA	West Indian	Antillean	Guape	208.0	10
Brazil	Pernambuco	CMA	West Indian	Antillean	Araqueto	190.0	10
Brazil	Amazonas	INPA	Amazonian	N/A	Anama	112.5	10
Brazil	Amazonas	INPA	Amazonian	N/A	Ere	112.0	10
Brazil	Amazonas	INPA	Amazonian	N/A	Aria	113.0	10
Brazil	Pará	MPEG	Amazonian	N/A	Uiara	100.0	10
Brazil	Amazonas	CPPMA	Amazonian	N/A	Acai	103.0	10
Brazil	Pará	BioMA	Amazonian	N/A	Neguinha	98.0	12

### West Indian manatees

The vocalizations of six Florida calves were recorded at ZooTampa in Florida, USA from 2005 to 2018. Recordings were made with a Cetacean Research Technology SQ26-08 hydrophone (sensitivity: − 169 dB re 1 V/µPa; sample rate: 48 kHz; resolution: 16 bit) (*n* = 5 calves) and an Aquarian H2A hydrophone (sensitivity: − 180 dB re: 1 µPa; sample rate: 48 kHz; resolution: 16 bit) (*n* = 1 calf) sampling to either a Sony PCM M-10, Sony DAT TCD-D8, or a M-Audio MicroTrack II Digital Multi Track Recorder.

Animals were routinely housed together at ZooTampa. To ensure calls were obtained from the same individual, calves were recorded under the following conditions: (1) when they were alone in a medical pool; (2) wrinkling of the nose was observed while a vocalization was being produced; and (3) the perceived level of loudness in relation to the hydrophone^19^. During sound production, manatees contract the muscles dorsal to the maxilla and caudal to the nostrils while maintaining them closed^35^. Such nasal cavity movements may have a role in sound modulation and filtering. Perceived loudness includes actively listening to calls as they are being produced and noting the location of animals in relation to the hydrophone. The closer a vocalizing animal is to a hydrophone, the louder the sound will be. When a call was produced, the call was annotated into a second, synchronized recorder. This happened when the calf vocalized within 1–2 m of the hydrophone.

Vocalizations from six isolated Antillean manatee calves were recorded from 1998 to 2018 at three locations: the Centro Mamíferos Aquáticos/IBAMA in Pernambuco, Brazil (*n* = 3): Dolphin Discovery, in the state of Quintana Roo, Mexico (*n* = 1); and the Caribbean Manatee Conservation Center at the Inter American University of Puerto Rico (*n* = 2). Recordings in Brazil were made with a Sony Walkman Pro WM-D6C Cassette Player (flat frequency response: 40–15,000 Hz ± 3 dB) and a Cetacean Research Technology 50Ca hydrophone (sensitivity: − 161 dB re: 1 µPa; sample rate: 44.1 kHz; resolution: 16 bits). Recordings of the two calves from Puerto Rico in 2011 were sampled with a SS03-10 *Sea Phone* directional hydrophone (sensitivity: − 169 dB re: 1 µPa; frequency range response: 20 Hz to 50 kHz) at a sample rate of 44.1 kHz in 16-bit resolution onto a Edirol R-44 digital recorder (frequency response 20 Hz to 40 kHz + 0/− 3 dB). The hydrophone was connected to two 30.5-cm suction cups, one placed on the throat and another on the nasal region caudal to the nares^35^. One calf recorded from Mexico was made with a Cetacean Research Technology 50Ca hydrophone (sensitivity: − 161 dB re: 1 µPa; sample rate: 44.1 kHz; resolution: 16 bits) sampling to an M-Audio MicroTrack II Digital Multi Track Recorder.

### Amazonian manatees

Vocalizations from six isolated Amazonian manatee calves were recorded at four institutions in Brazil from 1998 to 2019: Instituto Nacional de Pesquisas da Amazônia in the state of Amazonas (*n* = 3 calves); Museu Paraense Emílio Goeldi in the state of Pará (*n* = 1 calf); and Centro de Preservação e Pesquisa de Mamíferos Aquáticos of Manaus Energia, in the state of Amazonas (*n* = 1 calf). More details on calf recordings can be found in Sousa-Lima et al.^17,18^. Neguinha’s vocalizations were recorded at Biologia e Conservação de Mamíferos Aquáticos da Amazônia in the state of Pará, Brazil using a SoundTrap HF300 acoustic recording system (Ocean Instruments, New Zealand) at a sample rate of 576 kHz in 16 bits. All other manatees (*n* = 5) were recorded with a Cetacean Research Technology 50Ca hydrophone (sensitivity: − 161 dB re: 1 µPa: sample rate: 44.1 kHz: resolution: 16 bits) on TDK SA60 cassette tapes using a Sony Walkman Pro WM-D6C Cassette Player and digitized at a sampling rate of 48 kHz.

The procedures during the recordings of Florida and Antillean manatees in Puerto Rico were approved by the US Fish and Wildlife Service (LOA #63658B and permit number M791721-4). Protocols on live animals in Puerto Rico were approved by the Inter American University’s Institutional Animal Care and Use Committee. Recordings of manatees in Brazil were approved by the Brazilian Government and the Animal Behavior Society’s Animal Care Committee. All methods were performed in accordance with relevant guidelines and regulations as suggested in the ARRIVE guidelines.

### Acoustic analysis

**Figure 1:**
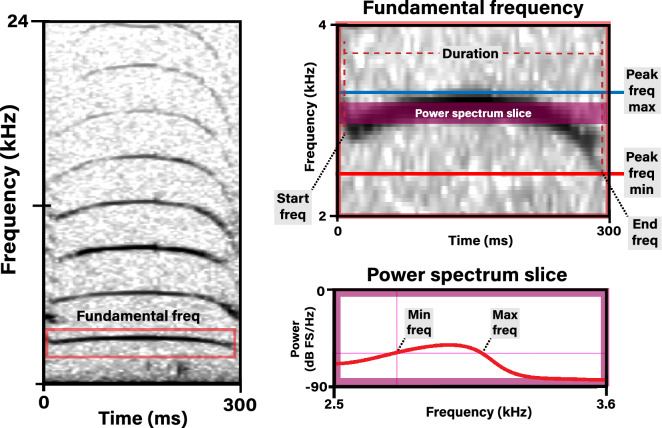
Schematics showing how the acoustic parameters of the vocalizations of manatee calves were extracted. The spectrogram on the left shows a single manatee call with the fundamental frequency of the call boxed in. The same box is expanded in the top right to show how duration (ms), frequency modulation (kHz) (peak frequency maximum minus peak frequency minimum), start frequency, and end frequency (kHz) were measured. The shaded area overlapping the call illustrates the power spectrum slice expanded in the bottom right showing the measurement of the power at the minimum and maximum frequencies (kHz) of the selected call. All measurements were conducted in Raven 1.6^39^. Spectrogram parameters: DFT: 512 point; Hamming window; 50% overlap.

To compare the acoustic parameters of the vocalizations of our three populations, we manually reviewed recordings by visually inspecting spectrograms to select high-quality calls for analysis. Spectrograms were calculated by fast Fourier transform (FFT) with a time resolution of 6.1 ms and a frequency resolution of 46.8 Hz (DFT: 1024 point; Hamming window; 50% overlap) in Raven Pro 1.5^39^ Calls were selected if they had a signal-to-noise ratio (SNR) ≥ 6 dB and if they were clearly discernible with no overlapping calls or noise in the spectrogram. The fundamental frequency (the first harmonic in a series) of each call was boxed using the selection tool to draw a tight selection box (in the frequency and time domains) around the contour of the first harmonic. Amazonian manatees tend to vocalize in notes, i.e., short duration calls produced in a bout^17^; thus, all notes were included in the selection boundary when making measurements of the fundamental. Eight standard acoustic parameters were measured from the fundamental frequency: duration (ms); center frequency (Hz); duration (ms); bandwidth (Hz); maximum and minimum frequency (Hz); start and end frequency (Hz); and frequency modulation (Hz) (defined in Table 2 and illustrated in Fig. 1). Maximum and minimum frequency was derived from the average power spectrum across the entire measurement box using a 10 dB threshold^40^. Variables were chosen to be comparable with previous studies^17,18^.

**Table 2:** Definitions of acoustic descriptors used in analysis of manatee calf vocalizations.

Measurement	Description
Duration (ms)	Derived by subtracting the start and end time from the selection boundary
Center frequency (Hz)	The frequency that divides the selection into two frequency intervals of equal energy
Bandwidth (Hz)	Difference between the maximum and minimum frequency
Maximum frequency (Hz)	Maximum frequency of the call measured from the power spectrum
Minimum frequency (Hz)	Minimum frequency of the call measured from the power spectrum
Frequency modulation (Hz)	Calculated by subtracting the peak frequency contour min frequency from the peak frequency contour max frequency
Start and end peak frequency (Hz)	Start and end peak frequency obtained from pitch tracking algorithm within the selection boundaries

### Statistical analysis

To determine if call variables differ across species, we performed a one-way multivariate analysis of covariance (MANCOVA) with species as a fixed factor and body length as a covariate. Calf call frequency and temporal variables were Box–Cox transformed to adjust their distribution to nearly normal^41^. For the purposes of this study, Florida and Antillean were treated as separate populations. Due to measuring several acoustic features on each call and performing multiple tests on the same data set, the chance of committing a Type 1 error increases. A sequential Bonferroni adjustment of 0.006 was used to reduce the possibility of an erroneous statistically significant result^42^. A test of significance between species was determined using Wilk's Lambda and the effect size was assessed with partial n^2^ statistics. A discriminant function analysis (DFA) was used to determine the call variables that best distinguished between species. We selected a regularized compromised discriminant method with lambda = 0 and gamma = 0.1 because the covariance matrices for the group compositions were significantly different (Box’s M = 448.32, df1 = 72, *p* < 0.001). The Kappa Index test was used to assess how well the discriminant function performed than expected by chance at a statistical significance level of 0.05^43^. The cross-validated method was used to calculate correct classification scores for the discriminant functions. Chi-square tests were used to evaluate the accuracy of the classification at a p-value of 0.05^41^. All calls from each species were treated as a group^17,18^. Prior probabilities were obtained from the number of calls used in analysis for each species. Although some variables were correlated, all variables were retained to minimize the loss of information^44^. All analyses were performed using SPSS version 28.0^45^.

## Results

### Interspecies variation in call variables

**Figure 2:**
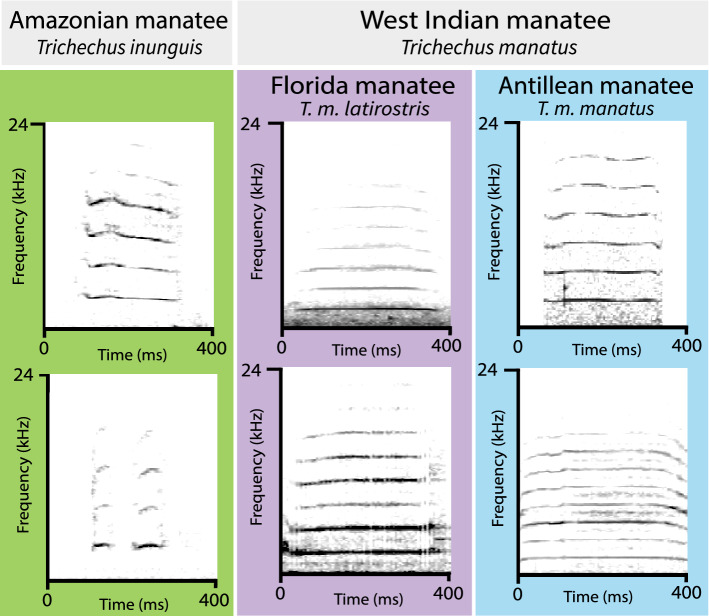
Spectrograms of vocalizations recorded from individual calves in this study from the Amazonian manatee (*Trichechus inunguis*) and the two subspecies of the West Indian manatee (*T. manatus*)—the Antillean manatee (*T. m. manatus*) and the Florida manatee (*T. m. longirostris*).

Florida and Antillean manatees (subspecies of the West Indian manatee) produced mostly tonal calls with hill-shaped contours (i.e., higher frequency modulation). Amazonian manatee calves also produced calls with frequency modulation, but it was not as accentuated as the hill-shaped calls observed in the Florida and Antillean manatees (Fig. 2). Amazonian calf calls contained one to three notes. The three smallest Amazonian calves produced calls that contained notes as well as the largest Amazonian manatee calf. Florida manatee calves produced calls that ranged in frequency from 2.25 to 3.42 kHz and call duration ranged from 0.089 to 0.371 ms. Antillean manatee calls had similar ranges to Florida manatee vocalizations for call duration (0.105–0.420 ms) and frequency range (1.87–3.96 kHz). The fundamental frequency and duration of Amazonian calf vocalizations ranged from 3.18 to 5.06 kHz and from 0.031 to 0.314 ms, respectively.

**Figure 3:**
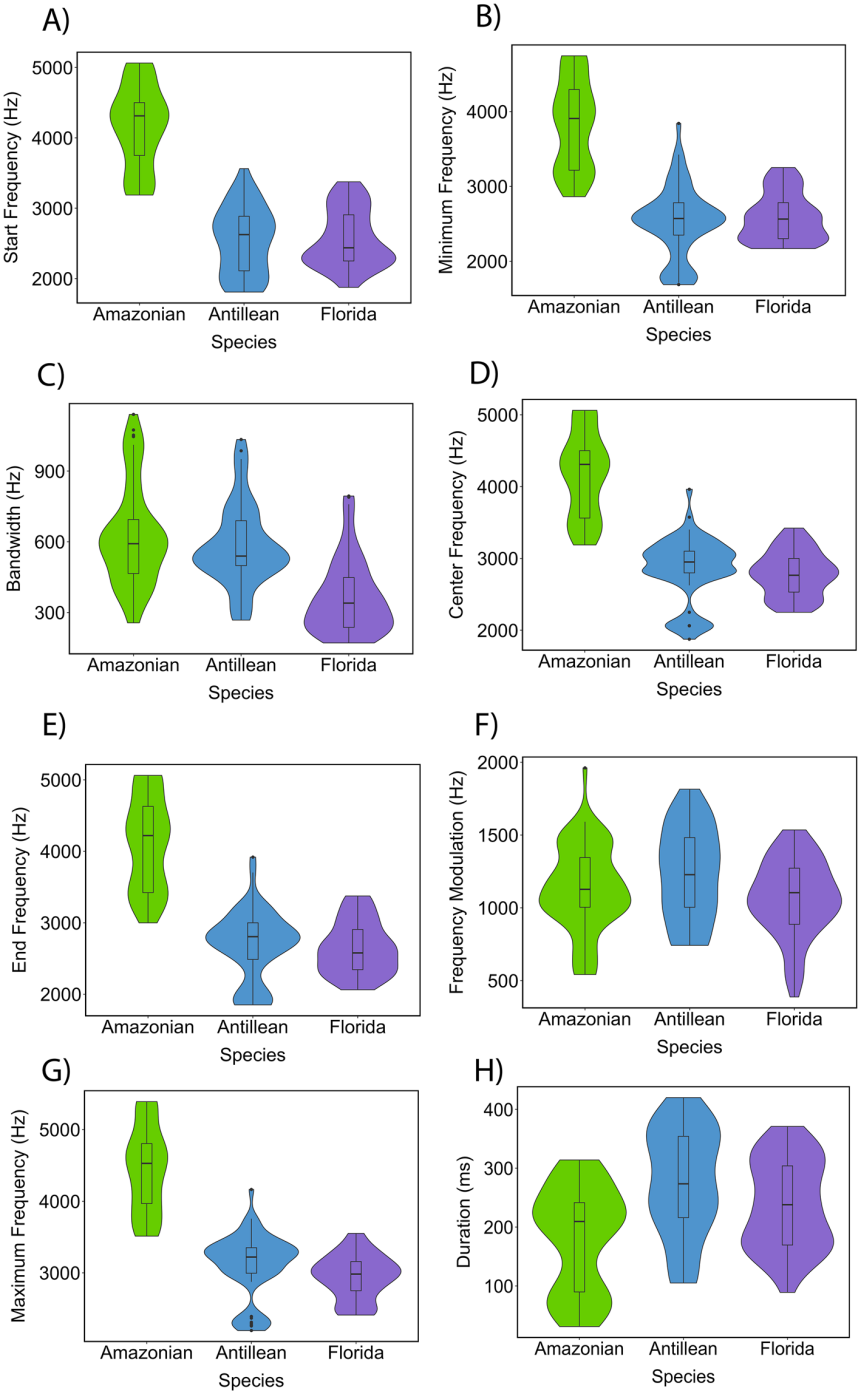
Manatees call frequency and temporal variation by populations. Box plots display medians, first and third quartiles, as well as standard error.

Amazonian manatees produced calls with shorter duration, higher start, center, minimum, end, and maximum frequencies compared to the Antillean and Florida manatees (Fig. 3A–H). After accounting for differences in body length, we found that Amazonian, Florida, and Antillean calves produce calls with acoustic features that are significantly different (Wilk’s λ = 0.21, F(16,340) = 24.53, *p* < 0.001). The multivariate n^2^ = 0.536, indicates that 53% of the multivariate variance of the call frequency and time variables is associated with the species.

The discriminant analysis found that Functions 1 and 2 predict the differences in call acoustic variables between species (Function 1 through 2 Wilk’s λ = 0.198, χ^2^ = 282.97, *p* < 0.001 and Function 2 Wilk’s λ = 0.678, χ^2^ = 67.81, *p* < 0.001). Based on the standardized coefficients, function 1 was related primarily to minimum frequency and bandwidth, and function 2 was related to minimum, center, and start frequency, and bandwidth. Discriminant function 1 explained 70.8% of the differences in call acoustic structure among species (eigenvalue = 2.43, canonical correlation = 0.84) and function 2 explained 32.1% of the differences (eigenvalue = 0.475, canonical correlation = 0.567). The overall number of calls correctly classified to species was 82.9% (Kappa = 0.726, *p* < 0.001).

### Differences between the West Indian (Florida and Antillean) manatee subspecies

**Figure 4:**
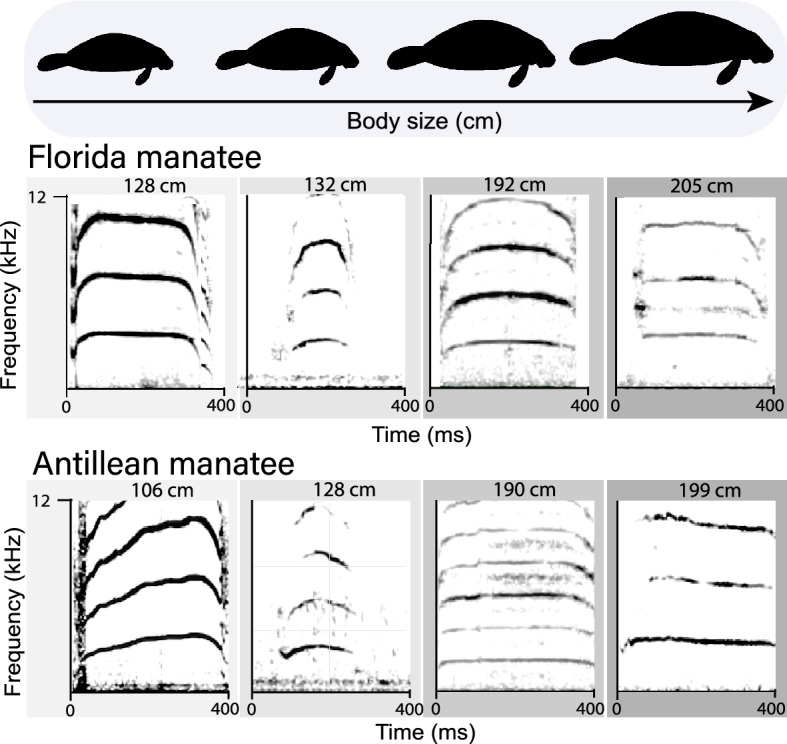
Ontogenetic changes from a hill-shape to a linear call contour of the vocalizations of the Florida manatee (*T. m. latirostris*) and the Antillean manatee (*T. m. manatus*). The Florida and Antillean manatee are subspecies of the West Indian manatee (*Trichechus manatus*),

We noted that the West Indian species had similar hill-shaped and linear call contours based on body size and differences in bandwidth as determined by the discriminant analysis. Frequency modulation and bandwidth can be correlated with body size^17,18^. Based on this, we performed an exploratory linear regression to predict if body length influenced frequency modulation and bandwidth across the two subspecies (Antillean and Florida) of the West Indian manatee. We used the mean values of each of the variables from each individual manatee calf across the two species^17,41^. Only frequency modulation was significantly correlated with body length (F (1,10) = 14.343, *p* < 0.004) with an *R*^2^ of 0.589. Frequency modulation decreased − 0.106 Hz with each increase in centimeter of body length, i.e., smaller animals vocalized with calls that tended to have a hill shape. As manatees grew in length, the hill shape appeared to flatten (Fig. 4).

## Discussion

Identifying interspecific variation in the acoustic characteristics at different ages provides insights into the influence of different selective pressures on the ontogeny and function of acoustic calls. The goal of this study was to investigate how the vocalizations of the two subspecies of the West Indian manatee (Antillean and Florida) and the Amazonian manatee varied between populations. All three populations show similar morphology of the vocal tract^35^, which would suggest similarity in call contours and frequency and temporal characteristics. However, Amazonian calves tended to vocalize in multiple notes as opposed to one continuous call. In addition, Amazonian manatee calves produced calls that were higher in frequency and shorter in duration than Florida and Antillean manatees. This suggests that interspecific differences observed in Amazonian calf calls may have evolved due to factors unrelated to morphology of the vocal tract and may be related to body size. There were few differences in frequency and temporal values between Florida and Antillean manatee calves. Finally, we noted a decrease in frequency modulation in Florida and Antillean manatees that may be influenced by ontogeny.

The smaller body length of the Amazonian manatee may have caused the higher frequency and shorter duration vocalizations than the Antillean and Florida manatees. Amazonian manatees have a maximum total body length of 300 cm^20^ and are approximately 85–105 cm at birth^46^. Animals with a smaller body size tend to produce calls at higher frequencies than larger animals^47^. The body length of the Amazonian manatee remains relatively stable through the first year and increases slowly thereafter^46,48^. In contrast, the West Indian manatee species grows exponentially faster over the first year^37,49^. Body length may also partially explain the differences in duration of calls between species, i.e., Amazonian manatee calves produced shorter duration calls. In terrestrial animals, the duration of calls tends to lengthen with an increase in body size due to maturational changes in lung capacity^50^. The variability in growth patterns may influenced the frequency and temporal characteristics observed in Amazonian manatees.

Body size may have also influenced call contours in Florida and Antillean manatees. Call contours in younger, smaller animals were hill-shaped and tended to flatten in older, larger calves in the Florida and Antillean manatees. Observations from this and prior studies^18,19,32^ suggest that the hill-shaped call is a stereotypical call produced by smaller calves. Changes associated with lowering frequency modulation are likely due to the lengthening of the vocal tract with age^17,18^. The flattening of the hill shape over time as the animal becomes independent of its mother is likely associated with the transition during weaning and less dependency of the calf on the cow. Manatees may undergo periods of crystallization like passerine birds or as speculated for northern elephant seals in which after a certain age the structure of calls and variability of vocal repertoire are fixed^51,52^. These age-specific stereotypical hill-shaped features in Florida and Antillean manatees likely evolved to aid cows in discriminating calves from adult conspecifics. Furthermore, individualized acoustic parameters within calf calls can be used in recognition by lactating mothers^17^. This provides additional evidence of sirenian cognitive abilities to discriminate acoustic signals.

In addition to similarities in call contour, comparisons of measured variables of the fundamental frequency of Florida and Antillean manatee calves showed few statistically significant differences. Antillean and Florida manatees are genetically distinct from one another^53^ and diverged from a common ancestor approximately 1.34 Mya with some evidence for intermixing between populations^54^. Another plausible explanation for shared vocal characteristics is that West Indian manatees have similar habitats. Similar habitats can influence convergence in acoustic signals between genetically distinct populations^55^. Geographical variation in the call types of other species of marine mammals suggest the divergence of call types and structural components of calls are a common byproduct of local adaptation^56,57^.

Signal adaptation to different habitats could also have resulted in the differences observed in the Amazonian calf vocalizations. Many animals are reported to adjust the frequency and temporal content of vocalizations to optimize propagation of signals in their respective habitat^58,59^. The Amazonian manatee is completely freshwater dependent^20^ as opposed to Florida and Antillean manatees who are distributed throughout freshwater, brackish and marine habitats^24^. Freshwater and seawater ecosystems vary in sound propagation loss. For example, freshwater systems exhibit less sound absorption loss because they have less salt content than seawater systems. This could facilitate larger transmission distances of high-frequency signals^60^. However, a recent study found that frequency bands between 0.5–20 kHz have been shown to propagate efficiently in freshwater, marine and brackish habitats for the Antillean manatee and in seagrass beds for the Florida manatee^59,61^. Furthermore, behavioral audiograms identified hearing capabilities ranging from 0.4 to 90.5 kHz with peak sensitivity at 16 to 18 kHz in Florida manatees^62,63^. Evoked potential studies conducted with the Amazonian manatee show best hearing sensitivity between 5–20 kHz going up to a maximum of 50 kHz^64^. The overlap in hearing and frequency bands that propagate best in freshwater, marine and brackish environments suggest that habitat may not be the driver for the observed differences in vocalizations between Antillean, Amazonian and Florida manatee populations.

Similarly, the captive environment also may have influenced frequency and temporal parameters of vocalizations. Isolating manatees for recordings could have caused stress to the individual. Florida manatees under condition of stress (while being captured for health assessments) increased duration and had more frequency modulated calls^32^. Some of the Florida manatee calves were not isolated for this study, yet duration and frequency modulation of measured calls were not different from isolated Antillean manatees. Amazonian manatees were isolated, yet their calls were still shorter and showed less frequency modulation than Florida and Antillean manatees. Furthermore, fundamental frequencies recorded from wild Florida calves were reported ranging from 2.5 to 4 kHz^19^ and overlap with the fundamental frequency of vocalizations from captive Florida manatees in this study. Therefore, we believe the captive environment did not influence our results.

Future research should incorporate longitudinal comparisons of call structure of individuals to evaluate how the hill-shaped call structure changes in association with age, throughout development, and into adulthood. This would require localization of calls of individually recognizable animals and easiest done in captive facilities including aquariums and rehabilitation centers. Acoustic tags^65^ placed on young individuals could also reliably follow the ontogeny of vocalizations during development. Playback of hill-shaped calls of calves of different ages to female manatees (with dependent calves or recently independent calves) could be used to test whether the decreasing frequency modulation of call structure influences the behavior of a mother manatee. For example, if the calls of their own calf (known to elicit a strong response during lactation^17^) are recorded at various ages stimulate the same level of reactions when played back to the mother.

## Conclusions

Our study provides evidence for interspecific variation in frequency and temporal parameters of manatee calf calls and in the ontogeny of call contours which is most likely related to body size. Amazonian manatee calves produce calls of higher frequency, shorter duration, and did not show the distinct hill-shaped contour used by both the Florida and Antillean manatee. Younger Florida and Antillean manatees tended to vocalize with a hill-shaped contour that appears to become more adult-like as it ages. Developmental changes in call structure seem to be absent in Amazonian manatees, corroborating previous evidence that strategies used by calves to call conspecific attention may be unique to Florida and Antillean manatees^17,18^. Our findings indicate Florida and Antillean calves retain call characteristics that make them distinguishable as calves from adults.

## .

## Data Availability

All data is available upon reasonable request to the corresponding author.
